# The Effect Of microbial Mats In The Decay Of Anurans With Implications For Understanding Taphonomic Processes In The Fossil Record

**DOI:** 10.1038/srep45160

**Published:** 2017-03-24

**Authors:** M. Iniesto, I. Villalba, A. D. Buscalioni, M. C. Guerrero, A. I. López-Archilla

**Affiliations:** 1 Department of Ecology, Universidad Autónoma de Madrid, 28049 Madrid, Spain; 2 Biogéosciences UMR6282, CNRS, Université Bourgogne Franche-Comté, 21000 Dijon, France; 3 Department of Biology, Universidad Autónoma de Madrid, 28049 Madrid Spain

## Abstract

The pattern and sequence of the decomposition of the Pipidae African dwarf frog (Hymenochirus *boettgeri*) is tracked in an experiment with microbial mats in order to explore soft tissue preservation over three years. Frog decay in microbial mats is preceded by rapid entombment (25–30 days) and mediated by the formation of a sarcophagus, which is built by a complex microbial community. The frog carcasses maintained a variety of soft tissues for years. Labile organic structures show greater durability within the mat, cells maintain their general shape (bone marrow cells and adipocytes), and muscles and connective tissues (adipose and fibrous tendons) exhibit their original organic structures. In addition, other soft tissues are promptly mineralized (day 540) in a Ca-rich carbonate phase (encephalic tectum) or enriched in sulphur residues (integumentary system). The result is coherent with a bias in soft-tissue preservation, as some tissues are more likely to be conserved than others. The outcomes support observations of exceptionally preserved fossil anurans (adults and tadpoles). Decomposition in mats shows singular conditions of pH and dissolved oxygen. Mineralization processes could be more diverse than in simple heterotrophic biofilms, opening new taphonomic processes that have yet to be explored.

Studies in taphonomy are essential to properly interpret the fossil record. Most of this research can be sustained through direct observation of fossil specimens, but experimental taphonomy has become a promising field. Taphonomic experiences are needed to understand soft tissue fossilization (i.e., decay and preservation). An efficient approximation is to explore how tissues decay and their potential for preservation by considering the embryonic nature and properties of their cells within a phylogenetic context^1^. Outstanding results of these experiments show that a delay in cell autolysis is relevant for exceptional soft tissue preservation^2^,^3^,^4^,^5^,^6^,^7^ and that each tissue has its own preservation bias. In other words, tissues can be selectively mineralized; this was tested in invertebrates and vertebrates^8^,^9^,^10^,^11^,^12^. In addition, several experimental approximations have developed an ecological scope, and they explore the interaction between microbial activity and the carcass, including the inner microbiota of decaying bodies^2^. For such an approach, soft tissue preservation is mostly the result of authigenic mineralization^8^,^9^. Most microorganisms that have been used in these assays correspond to populations (from sea or lake sediments refs 8,9,13, 14, 15, 16) that grow by forming simple non-structured and labile biofilms, which are dominated entirely by heterotrophic microorganisms. However, our experiences and results from studying the effect of microbial mats on decay and preservation appraise a more complex community. Studies on mats are scarce, although it has long been assumed that mat communities are related to exceptional preservation^14^,^17^,^18^,^19^.

Microbial mats are stratified communities with high diversity that are mainly composed of primary producers (photo- and/or chemoautotrophic) and consumers/decomposers (heterotrophic)^20^. The occurrence of a food web with an interconnected energy flow and an almost closed cycle of nutrients leads researchers to consider these communities as an ecosystem on their own^21^. In phototrophic microbial mats, most of the physicochemical properties are conditioned by the vertical gradient generated by light penetration and the metabolic activity of microorganisms^20^,^22^. Based on the inherent properties of mats, some authors believe that these communities could play a key role in fossilization, for example, in obrution (i.e., quick microbial growth that enables rapid coverage of carcasses)^18^,^23^. Experiments show that the rapid growth of mats over animal carcasses promotes the formation of a protective sarcophagus^24^. This sarcophagus favours the formation of impressions and replicas at the carcass-mat interface. Fossilization by microbial mats also includes authigenic mineralization by lithifying (e.g., refs 25, 26, 27, 28). Experiments conducted with fish demonstrate the authigenic formation of a poorly crystalline Mg-silicate phase in relation to the decay of carcasses^29^.

This study describes the pattern and sequence of decomposition of the African dwarf frog (*Hymenochirus boettgeri*) in microbial mats (see *Experimental taphonomy* in the Materials and Methods). Our aim is to decipher the potential for exceptional preservation by exploring the permanence of cadaver integrity and long-term tissue preservation (several years). Previous studies with modern fish have partially reached these objectives^24^,^29^,^30^. In fact, in the case of vertebrates, experimental taphonomic approaches have been frequently conducted with fish (e.g., refs 31 and 32), with a few studies on other groups (e.g., birds ref. 33). Our outcomes also help to improve the understanding of how the fossilization of exceptionally preserved vertebrates occurred. One example is the adults and tadpoles of anurans from the famous locality of Libros (Miocene, Spain), where the carbonaceous outline of the body and several inner soft tissues (e.g., myofilaments, sarco-lemma and circulatory vessels) have been preserved^34^,^35^. Exceptional fossil vertebrates are represented in many famous deposits of different geological ages and environmental conditions, such as Las Hoyas (a carbonatic lacustrine deposit from the Lower Cretaceous in Spain)^36^,^37^,^38^, the Crato Formation (hypersaline carbonatic waters from the Lower Cretaceous in Brazil)^39^,^40^, the Green River Formation (intermountain lakes from the Eocene in the USA)^41^, Río Pichileufú (volcanic caldera lake from the Eocene in Argentina)^42^, and Riversleigh (limestone caves formed during the Cenozoic in Australia)^43^. Although fish represent the most abundant fossils in many of these exceptional deposits, denominated Konservat Lagerstatten^44^, the aquatic fauna could also comprise amphibians (anurans, urodelans, and different extinct groups), as well as reptiles, birds and mammals, which are inhabitants of more terrestrial milieus. All of these fauna fossilize exceptionally, and taphonomic analyses in some of these localities indicate that bacteria influence the exquisite preservation of soft parts.

## Results

### Taphonomic changes

**Figure 1:**
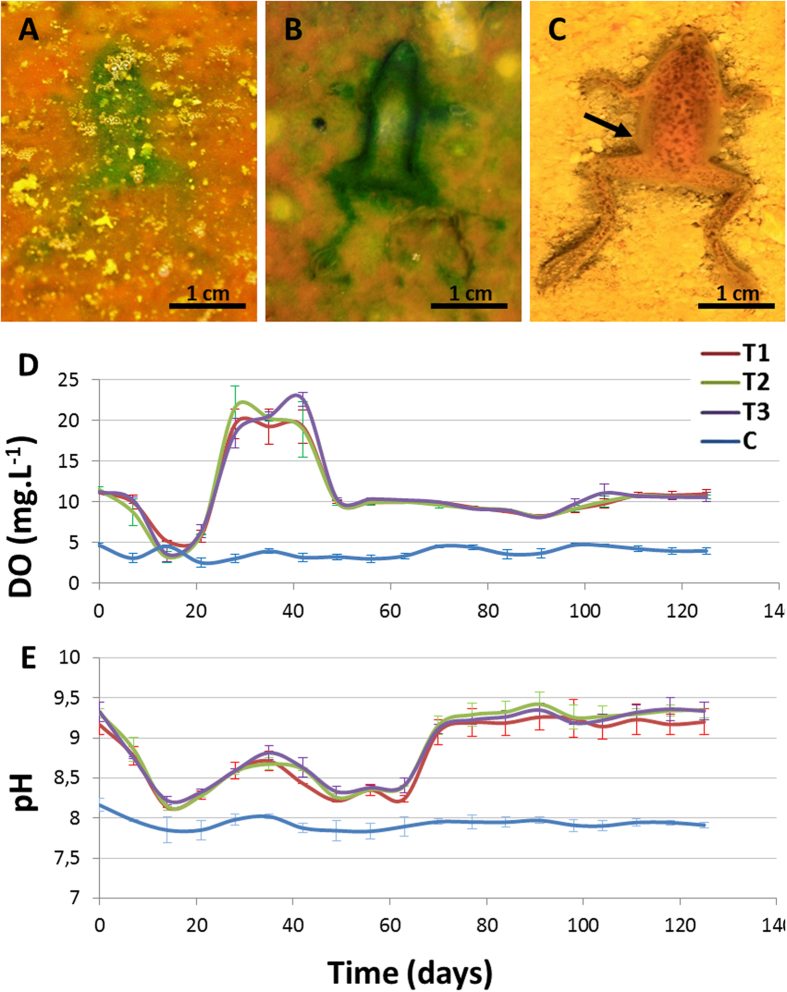
Taphonomic alterations of frog carcasses over a mat. (**A**) and (**B**), outlines of removed frog bodies, which are greened by local stimulation of the phototrophic mat populations. In (**B**), the thickness of the frog contour is due to incipient sarcophagus formation. Experiments in (**A**) and (**B**) were conducted at day 3 and day 7, respectively. (**C**) Frog in the control showing a darkened outline of the sediment that is in direct contact with the body (day 7). (**D**) and (**E**), variations of DO and pH in water from the tanks with mats (T1-3) and the control (**C**) over the course of the experiment.

The microbial community, especially the oxygenic photosynthetic populations, were stimulated with the placement of frog carcasses on the upper layer of the mat. This layer transformed to a dark-green colour as a result of fast-growing increase of the mat during the first week (Fig. 1A and B). Increased mat activity resulted in environmental changes in the water column. Dissolved oxygen (DO) and pH were very similar in the three mat tanks during the course of the experiment but significantly differed from the control tank values. In the control tanks, DO remained stable and low (below 5 mg·L^−1^). In contrast, mat tanks exhibited much more variation. An initial 14-day decrease (Fig. 1D) was followed by an increase of up to 15 mg·L^−1^ between days 14 and 42. After day 42, DO recovered the baseline values (around 10 mg·L^−1^) and continued to be almost constant over the following weeks. Similar variations were observed in pH (Fig. 1E); carcasses were laid on the mat surface, and the water column was acidified from 9.3 down to 8.1 at day 14. Subsequently, the pH increased slightly (to 8.7) on day 35 and increased again after day 63, reaching the initial values. After the last rise, the pH remained steady until the end of the measurements. Regarding the controls, the pH was more stable and lower (near 8) than in the mat tanks (Fig. 1E).

**Figure 2:**
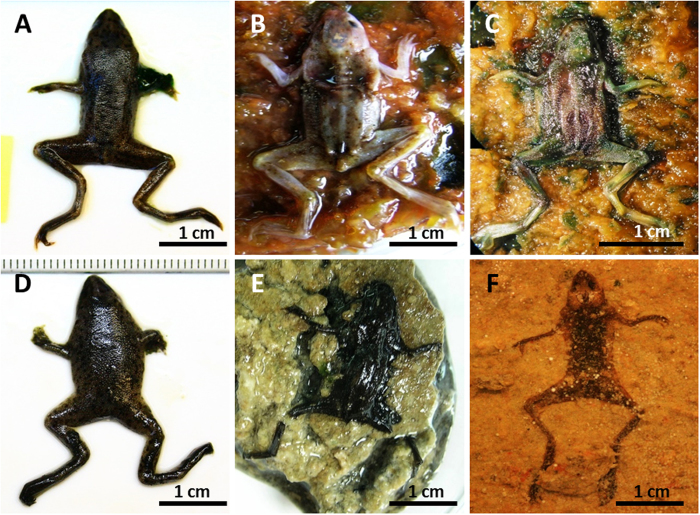
Composition showing the taphonomic alterations of carcasses during the experiment. (**A**–**C**) frogs on mat; (**D**–**F**) frogs on sediment. (**A**) and (**D**) Carcasses after day 15 on the mat and sediment, respectively. (**B**) Frog removed from the microbial envelop at day 120, demonstrating complete articulation and preserved soft tissues (eyes, skin, midbrain). (**E**) Decayed frog in the control. (**C**) Frog removed from the sarcophagus after 240 days. The carcass is still articulated. (**F**) Massive decay at day 240, showing a dark shadow over the sediment.

**Figure 3:**
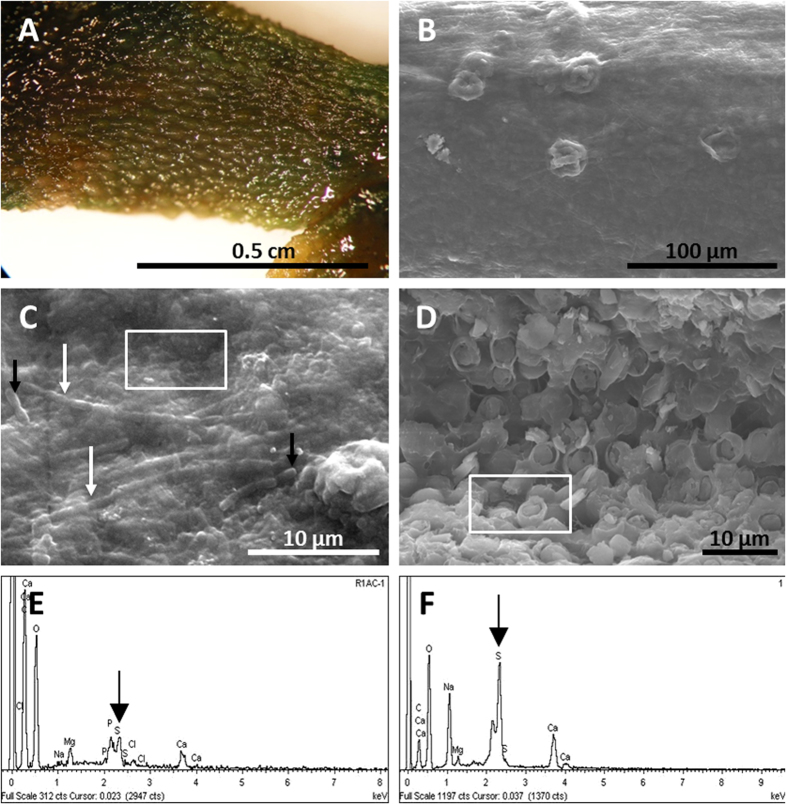
Frog skin preservation. (**A**) Skin warts in the hind-limb at day 240. (**B**) SEM warts (day 240). (**C**) SEM image of the microbial veil of the skin, after removal of the microbial sarcophagus, made by cells (filaments – white arrow, and bacilli-like – black arrows) and EPS after 540 days. (**D**) SEM of adipocytes preserved beneath the integument at day 1080 for the frog in the mat. (**E**) and (**F**), EDXS spectra recorded at the areas highlighted in (**C**) and (**D**), respectively. Black arrows point to the pic corresponding to S.

The general changes observed and the integrity of the corpses on the mats and for the controls were compared (see *Experimental taphonomy* in the Materials and Methods section below). Soon after positioning the anurans on the mats (25–30 days), the carcasses were trapped by bacterial filaments from the upper cyanobacterial layer, building a microbial sarcophagus (Supplementary Fig. S1A–F) that covered the corpses henceforth, while for the controls, the carcasses decayed directly over sediments. At day 15, frogs on the mats were fully articulated with cohesion (Fig. 2A). In comparison, for the controls, the frogs showed moderate symptoms of decay, although they were articulated (Fig. 2D). In fact, the sediment around the frogs in the control darkened beneath the body (Fig. 1C) as a consequence of sulphide production from the decay that reacted with the Fe in the sediment^32^,^45^. These differences increased until day 30, once the skin of the frogs on the sediment was pierced and the corpse became disarticulated during manipulation. In contrast, frogs covered by the mat maintained their original integrity, allowing complete handling during data collection. Although the frogs on the mats significantly flattened on day 120, they maintained their original consistency (Fig. 2B). In contrast, controls were so decayed that measurement and manipulation were impracticable (Fig. 2E). At day 240, the control frogs were nothing more than an organic dark spot marking the surface and edges of the animal (Fig. 2F); at this stage, slight movement provoked disconnections. This strong decay contrasts with frogs laid on mats (Fig. 2C), where the limbs were completely articulated at day 240. The skin remained elastic with its original texture full of warts (Fig. 3A). At higher magnification, integumentary integrity and scattered verrucae were confirmed (Fig. 3B) after 540 days (1.5 years). When the microbial sarcophagus was removed, residual small filamentous and bacillary microorganisms were adhered to the skin. The size and shape of these microorganisms were similar to those described on the surface of flies and fish^30^ (Fig. 3C). After day 1080 (3 years), the skin persisted unbroken and covered the entire skeleton. In fact, adipocyte-like cells were detected in the skin laceration (Fig. 3D). It is notable that EDXS analysis of the surface of the skin showed enrichment in sulphur and calcium between 540 and 1080 days (Fig. 3E–F).

**Figure 4:**
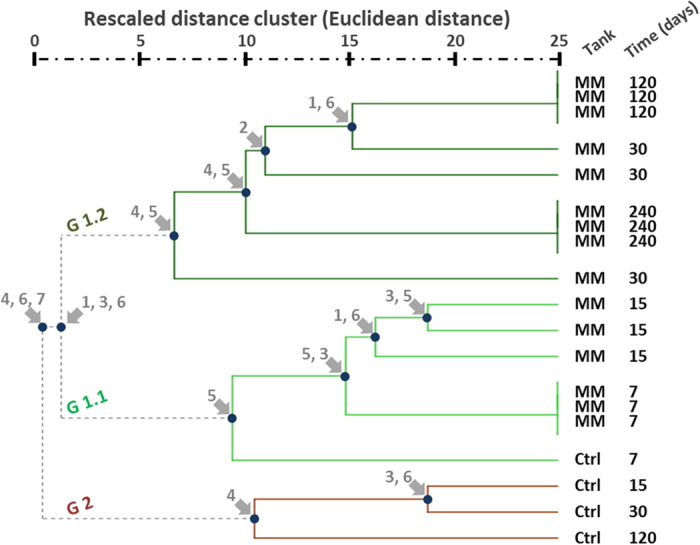
Dendrogram obtained by hierarchical cluster analysis, which shows the similarity of the sample carcasses according to the observed phenotypic changes (MM: carcasses over mat; Ctrl: controls, carcasses over sediment). At each major node, the variable(s) responsible for the division is noted. Initially, both control and mat samples swelled (first 7 days). During this first week, decay of the carcasses in the mat and control tanks was similar. After this early stage, controls began to decay rapidly, leading to a grouping of the remaining control samples in a separate cluster. Thus, the presence of mats clearly influenced the sequence of decay. Controls presented at day 15 showed loss of skin rugosity (likely pierced), high disarticulation, and deformation. During the swelling, observation of the linea alba was difficult. In addition, a thin heterotrophic veil grew rapidly over the controls, demonstrating active decay. Mat samples showed neither deformation nor disarticulation along the experiment, and a thick layer of mat covered the bodies. 1: swelling, 2: colour, 3: red colour, 4: linea alba, 5: EPS, 6: skin, 7: articulation.

Continuous tracking of qualitative variables (see *Experimental taphonomy* in the Materials and Methods section below) provided a detailed description of the decay pattern. Acquisition of data resulted in the generation of a data matrix for the Hierarchical Cluster Test (Supplementary Table S1). Advanced decay of frogs on sediment (controls) did not allow for data collection after day 30 because manipulation would lead to specimen break down. The resulting dendrogram (Fig. 4) showed two major clusters: one including samples from the mats and controls at day 7 (Fig. 4G1) and the other grouping controls from day 15 (Fig. 4G2). Both were clearly defined by the decay state. The G2 cluster, formed by controls, corresponds with moderate to extensive decomposition. The G1 branch is completing divided into two other minor clusters. The first one (G1.1) grouped samples with none or subtle damages, explaining the inclusion of the day-7 control that did not show remarkable decay yet. The second one (G1.2) included the remaining samples on the mat. These groups were congruent with the outcomes of decay monitoring. Morphometric parameters showed that the body size varied over time. Frogs decaying on mats had an extensive loss of volume (up to 50% for thigh and abdomen thickness), but statistical tests showed that this loss was significantly higher in controls. These differences increased from day 15 onwards (Supplementary Tables S2 and S3). In samples with the mats, the loss of volume seemed to correlate with the type of variable measured (see plots in Supplementary Fig. S2).

### Long-term tissue durability

The outstanding preservation of frogs laid on mats made experimental manipulation possible; hence, the removal of carcasses after four months was possible without damaging their anatomy (Supplementary Fig. S3). The completeness of the body was due not only to the protection afforded by the sarcophagus that maintained the skin in good shape but also to the preservation of different tissues related to the musculoskeletal system (Supplementary Fig. S4A–F). Despite the mat having direct contact with the frog skin, epithelial integrity prevented the entrance of cyanobacteria filaments inside the body. This system provides form and stability to the body. The frogs on the mats (from 540 to 1080 days) have preserved skin, skeleton, muscles, different types of connective tissues, components of the haematopoietic system, and the nervous system.

**Figure 5:**
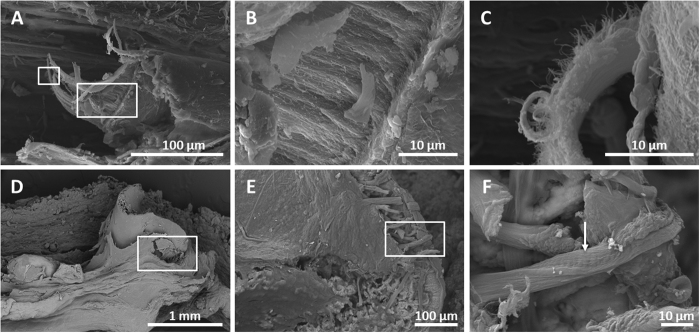
SEM photos of muscle (**A**–**C**) and connective tissue (**D**–**F**) in the frog in the mat at day 1080. (**A**) Femoral muscle that was ripped during the preparation shows different layers that have been magnified in (**B**) and (**C**). (**D**) Femoral knee articulation of a frog inset in the box. (**E**) Fibrous fibres of tendons from the same area, magnified in (**F**). The arrow highlights the striping of collagenous fibres.

Bones are preserved even after 3 years (see Supplementary Fig. S5). However, the bones of the frogs on sediment became completely disintegrated by this time. Femoral musculature was observed beneath a lacerated piece of skin (Fig. 5A–C). Different types of connective tissue, such as adipocyte cells (of approximately 5 μm), which are special connective adipose tissue of the organized fatty tissue underlying the dermis (Fig. 3D), were notable. Similarly, tendinous fibres (i.e., fibrous connective tissue) disposed in filaments with a distinctive striping are consistent with collagen that was recorded at the knee joint (observed in 1080-day specimens; Fig. 5D–F).

**Figure 6:**
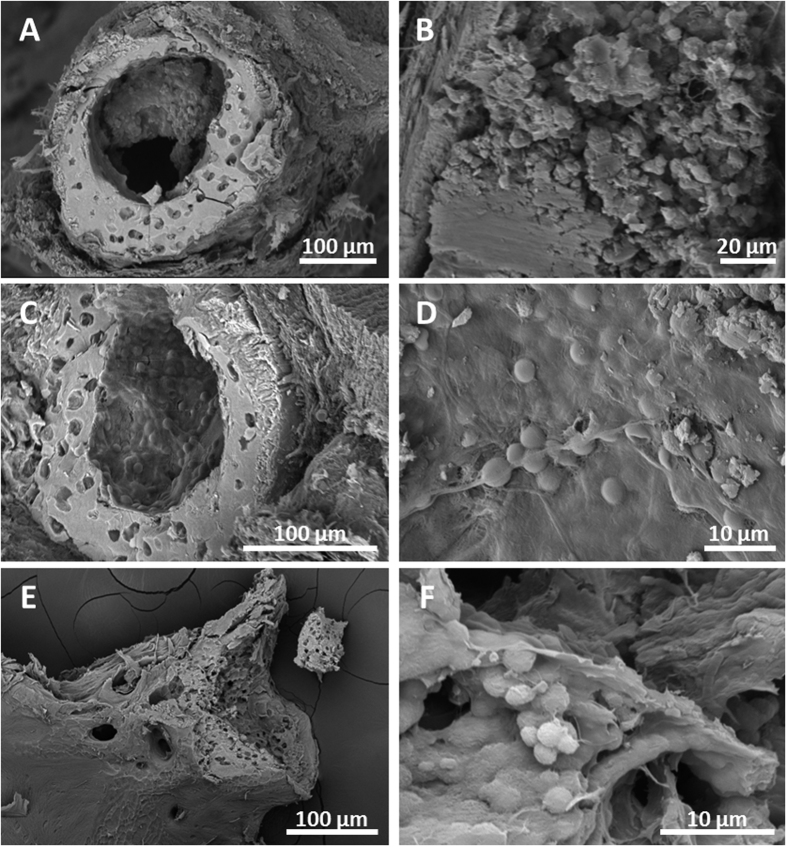
SEM photographs of bone preservation in the mats. (**A**) Section of the central portion of the femur after 1080 days, exhibiting the periosteum, the osteocyte lacunae, and exceptional preservation of the fatty marrow at the medullar cavity, magnified in (**B**). (**C**) Section of the tibio-fibula (at day 540) showing the periosteal bone with osteocytes lacunae and the haematopoietic tissue in the medullar cavity. (**D**) Detail of (**C**) showing the organic coat with haematopoietic marrow cells. (**E**) Sectioned vertebra (at day 540) exposing the tissue at the articular surface. (**F**) Detail of haematopoietic cells filling the vertebra.

The bone marrow was observed in bone sections in 1080-day specimens. Sections of the central portion of the femur (Fig. 6A) allowed for observation of an amorphous organic residue (Fig. 6B). This organic residue likely corresponds to the fatty fraction marrow, i.e., the yellow bone marrow. In addition, the haematopoietic system was detected in sections performed in the tibio-fibula and vertebra after 540 days (Fig. 6C and E respectively). Cells embedded in a mesenchymal tissue are frequently observed inside the bone cavities (Fig. 6D and F). According to their position, size (approximately 4 to 6 μm) and round shapes (sometimes concave), these cells were identified as erythrocyte-like (marrow cells) in the red bone marrow.

**Figure 7:**
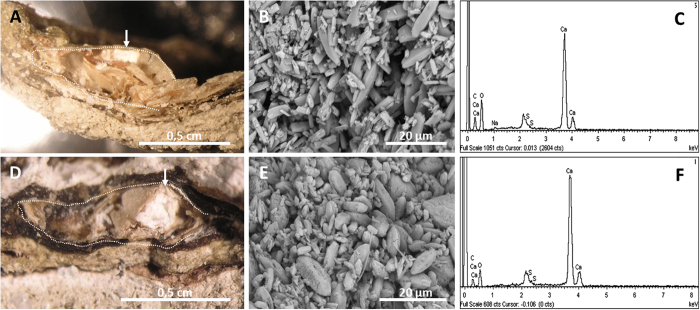
Preservation of the brain of the frog in the mat. Sagittal section of the skull at day 540 (**A**) and at day 1080 (**D**) with a binocular loupe. The line delimits the skull contour, and the arrow points to the tectum space. SEM photograph showing the calcite crystals in (**B**) and (**E**). (**C**) EDXS spectrum of carbonate in (**B**). (**F**) EDXS spectrum of minerals in (**E**).

Despite few taphonomic alterations, the eyes and brain were conserved at day 120 (Supplementary Fig. S6). An unexpected observation was the preservation of a part of the midbrain-the tectum (i.e., optic lobes). White hardened deposits were clearly recognisable inside the skull at 540 days (Fig. 7A and D for 1080 days). The frog midbrains (two cases) were transformed into calcium carbonate crystals (Fig. 7B and E, and the corresponding EDXS spectra Fig. 7C and F).

## Discussion

This experiment consisted of a set of systematic observations of frog carcasses placed over microbial mats and sediments for three years. In these taphonomic experiments, we tracked and measured the decay and preservation of the corpses as a consequence of the metabolic changes exerted by the microbial mat. The results provided data on the decay sequence, the microenvironmental conditions (pH and DO), and the influence of the mat on soft-tissue durability. The main result was that frogs in microbial mats showed a significant delay in decay, and the body maintained articulation over the years, replicating other results obtained with fish^29^,^24^,^46^. Bodies in control tanks showed fast decomposition, starting with a loss of integrity around day 30. These observations were supported by results from the Hierarchical Cluster Analysis, which separately grouped frogs from the control tanks and mats, demonstrating a significant level of dissimilarity. This is congruent with the results of a similar analysis performed with fish in mats, where samples were also clearly grouped according to mat and sediment^24^. Differences between the samples in the mat and control groups are also significant with regard to metric measurements. The initial size reduction came after an early swelling of the bodies, which is a typical forensic phase that has been described in taphonomic experiments, likely driven by the endogenous microbial decaying populations^47^. During this short period (7–15 days), the decay of carcasses in the mat and control tanks was similar; likewise, the first control (day 7) was grouped in the cluster with the mat samples. After this early stage, controls began a rapid decay that led to complete disarticulation at days 40–45, while carcasses on the mat remained articulated all along the experiment (1080 days).

### Decay

Autolytic activity (i.e., the process whereby lysosomal enzymes are released, provoking tissue degradation^3^) should have occurred in the cadavers’ tissues deposited in both the mats and control tanks during the first days of experimentation. The results showed a drastic reduction in oxygen concentration and pH during the first 14 days, and this was quite notable in tanks with microbial mats (Fig. 1). These changes in pH and DO denote the heterotrophic activity of decomposers that consume oxygen and acidify the system by releasing acid compounds during decay. However, the water column did not become fully anoxic or acidic in any of the cases. These results do not match with a fully acidic and reductive environment, as described in experiments on *Artemia salina*^2^ and sea urchin embryos^4^,^5^, which is required for the blockage of autolysis and, hence, a delay in decay^4^,^6^,^7^. In our assay, the environmental changes were controlled only in the water column, not inside the frog bodies. However, in a former experiment with fish, environmental conditions both inside and outside of the corpses were recorded by using microsensors. The results demonstrated that the inside of the fish had acidic and reductive conditions only during early decomposition (first week)^46^. Although autolytic blockage should have played a role in delaying the decay of frogs during the initial phase of decay, frogs in the mats showed considerable differences in preservation in relation to the controls after the initial phase. In the tanks with microbial mats, the photoautotrophic cyanobacteria increased in growth due to the nutrients released during early decay and the subsequent rise of oxygen concentration and pH. This environmental change, which was distinct from the initial environment, still favours preservation. Therefore, the results suggest that the main factor driving the delay in decomposition is the nature of microbial activity, not the autolytic blockage. In addition, the processes in the mat tanks led to fast coverage of the bodies in approximately 20 days and subsequent formation of the sarcophagus.

Experimental taphonomy of preservation shows that decreases in pH and DO are also crucial for biomineralization. The experiments with heterotrophic microbial communities link the occurrence of a mineralized coating of calcium carbonate^15^ and/or calcium phosphate^16^ in invertebrate eggs to these environmental conditions. Other tests performed with adult shrimp exhibited a drop in pH that inhibits the precipitation of calcium carbonate in favour of calcium phosphate in and over soft tissues^48^. These bioprecipitates occurred within one to four weeks and connected the microbial activity with the generation of anoxic conditions and a decrease of pH.

Our results showed that pH and DO were stabilized after seven weeks, returning to the initial values of basic pH and high oxygen concentrations. Nonetheless, no calcium phosphate precipitates were observed during the initial phase with decreased pH and DO, contrasting with the results of heterotrophic simple biofilm experimentation. However, we found that part of the midbrain of the frogs transformed into carbonate (see the section Soft-tissue preservation and fossils). This occurrence of carbonates could be explained by the saturation of Ca in water and the high pH^27^. Moreover, these conditions could probably be enhanced by the introduction of the limestone layer under the sediment in both mat and control tanks. This kind of precipitate, however, has only been detected in the presence of microbial mats. Thus, differences observed in mineral precipitation between experiments with mats and those performed with simple biofilms can be explained by variations in the metabolic activities. In the absence of mats, chemical conditions for mineralization are generated by heterotrophic decomposer populations^49^. Thus, the preservation processes must be rapid in order to conserve soft tissues. In mats, microenvironmental conditions are mostly determined by the autotrophic photosynthetic populations of the upper layers, and the continuous metabolic activity of the microbial community significantly delays body decay. In consequence, mineralization occurs more leisurely. Furthermore, the complexity of community metabolism introduces more variants to mineralization. In this sense, although it is broadly accepted that acidity and anoxia are necessary for calcium phosphate precipitation, these kinds of minerals can also occur under mat conditions according to the mineralogical data^50^,^51^. The variation in mineralization types seems to provide new and previously unknown evidence, for instance, the appearance of a Mg-rich silicate phase replacing the hydroxyapatite of fish rays and scales^29^. This amorphous silicate-phase has been recently described as a good carbonate precursor in the course of microbialite formation^52^. In addition, it has been recently reported that precipitation of silica during early-stages of fossilization can enhance preservation^53^,^54^.

### Soft-tissue preservation and fossils

Our frog samples had skin that was neither pierced nor torn after 1080 days, contributing to the maintenance of an articulated body. In fact, the state of preservation of the integument was so remarkable that the original external morphology of warts was still visible after 1.5 years. In addition, our experiments showed local enrichment of calcium and sulphur. Such an increase could explain the occurrence of gypsum on the preserved skin of adult frogs in the Miocene locality of Libros^34^. Furthermore, recent species of the family Pipidae are pharmaceutically well known for the antimicrobial activity of their skin secretions^55^,^56^. This peculiarity of the anuran skin could act as a factor to enhance skin preservation in frogs, as the frogs in the mats showed a completely conserved skin in relation to the controls. It is remarkable that the broad fossil record of anuran tadpoles shows preserved, articulated individuals with body outlines, as well as other soft tissues, such as cranial nerves, blood vessels and eyes^57^. Notoriously, the abundance is dominated by pipoid tadpoles^57^, which are in the family Pipidae that includes the African dwarf frog (*Hymenochirus boettgeri*) used in our experiment. This exceptional preservation is preceded by the formation of a microbial sarcophagus embedded in the EPS matrix, made of thick outer cyanobacterial filaments and small filamentous or slightly bacillary cells towards the carcass^30^. This protective sarcophagus has been described in fossils, such as the Lower Triassic frog from Gogolin (Poland)^58^.

The conserved connective and muscular tissues are fibrous tendons and muscles, and these were still perfect after one year in our experiment. These fibrous connective and muscle tissues are not rare in exceptional fossils (e.g., refs 44,59,60); these tissues have been characterized in frogs and salamanders as organic remains preserved as calcium phosphate^34^ or as sulphur-rich residue^61^. Not so common is the maintenance of haematopoietic cells. This tissue is extremely labile^62^ and rarely preserved in fossils. In the experiment, the erythrocyte-like cells, which coated the inner face in some bones, indicated the resistance of the haematopoietic tissue to decay, which persisted up to 3 years in several specimens. An analogous preservation was identified in 10% of adult frogs from the Miocene Konservat of Libros^63^. Recently, the occurrence of erythrocyte-like cells, similar to those observed in our experiment, were detected in the bones of different groups, such as theropods^64^, testudines^65^ and neornithischians^66^. These outcomes suggest that organic preservation of this type of tissue can occur more frequently than previously expected^64^,^67^.

Finally, early mineralization of the optic lobes by carbonates was unexpected because this Ca-rich precipitate was not widespread in the carcass or throughout the mat and only found in the mid-brain. This spatial limitation was not supported by the physicochemical evidence because when pH and DO were tracked using microsensors inside and around carcasses in the microbial mat, their values were equivalent^46^. Therefore, differences in mineralization could show that several soft tissues are more likely to be preserved^68^. Likewise, similar carbonate mineralization of the brains of frogs have been reported in the fossil record of tadpoles and adult frogs from Libros^34^,^35^ and from the Early Cretaceous limestone deposit of Las Hoyas (see Fig. 4A–B in^69^).

These types of studies help to reveal the taphonomic bias generated during decay, which is essential for interpreting the fossil record^70^. The decay in microbial mats is characterized by rapid entombment of the carcass (25–30 days) and mediation by a sarcophagus, which is a storage area rich in EPS and built by the complex microbial community. Metabolism of mat populations seems to be crucial for understanding the environmental changes of pH and DO. Notwithstanding, the preservation in mats is not tightly linked to acidic and reductive environmental conditions, which occur in heterotrophic simple biofilms. Our results show that frog corpses maintain a variety of soft tissues for years, whereby labile organic structures have greater durability within the mat. Cells maintain their general shape, and tissues show their original organic structures, denoting an inhibited decomposition. In addition, other soft tissues become mineralized (encephalic tectum) or rich in sulphur residues (skin). The microbial mat community acts as its own ecosystem. Thus, mats do not decay and instead continue dynamically by integrating the carcass decay into their biochemical cycles. Therefore, mineralization processes occur longer over time and may be more diverse compared to that of heterotrophic simple biofilms, thus presenting new taphonomic processes that have yet to be explored.

## Materials And Methods

To achieve the objectives described in this study, 36 bodies of frogs were placed on microbial mats grown in the laboratory or on sediment without mats as controls. All the experimental protocol was checked and approved by the authorized body of the government of the Comunidad de Madrid (CEI-UAM, i.e., Clinical Research Ethics Committee of the U.A.M.: http://www.uam.es/otros/ceiuam/), which evaluates protocols and projects including experiments with animals. In addition, all methods were performed in accordance with relevant guidelines and regulations.

### Microbial mats origin and growth in laboratory

Samples of the microbial mat came from “La Salada de Chiprana” (Zaragoza, Spain). This shallow lake is a permanent system of endorheic origin in a semiarid region of the Ebro river depression (Aragon, NE Spain). Its waters are hypersaline (30–70‰). The bottom of the lake is colonized by microbial mats from the shoreline to a water column depth of 1.5 m^71^, which is favoured by extreme physical and chemical conditions. Additional details concerning the physicochemical and biological characteristics of this site can be found in Vidondo *et al*.^72^, Jonkers *et al*.^73^ and Ludwig *et al*.^74^.

Microbial mats were grown in the laboratory under light and temperature controlled conditions. Tanks measuring 40 × 20 × 20 cm were filled with a 2 cm layer of sediment from the lake over a base of limestone of 2–3 cm. This bed was included in order to reproduce the natural environment of elements and sediment particles, which is rich in Ca and carbonates. This layer also acts as a buffer to compensate for the reduced pH that results from decay. Four tanks were used: three with microbial mats and one without a mat as a control. Soon after collection, the microbial community samples were ground using a basic mixer (T25 IKA Labortechnik), and the resulting mixture was spread equally over the corresponding sediment in three of the tanks. Tanks were filled with water from Chiprana until the column had a depth of approximately one centimetre. Previous experiments^29^ verified that the presence of this limestone layer did not significantly influence the chemical composition of the water column. The Ca, carbonates, sulphate and magnesium concentrations at the end of the experiment were similar to those observed at beginning of the experiment (detailed water analysis can be found in^29^). Dissolved oxygen (DO), pH, conductivity and temperature were monitored using specific probes (WTW Oxi 315i/SET, pH 3110 SET 2, 2C10-0011 and Cond 330i, respectively; temperature was measured with the oximeter) during the course of the experiment. Sterilized deionized water was added to tanks periodically to avoid strong variations in water level and salinity as a result of evaporation. Conductivity and temperature were relatively constant during the experimental times (mean 50.1 ± 2.3 mS·cm^−1^ and 23 ± 0.5 ºC, respectively). Tanks were illuminated by a 50 W halogen lamp (Osram Decostar 46870WFL), except for the control tank, which was kept dark to prevent the growth of phototrophic microbial mats from forms of resistance present in the sediment. The illumination intensity of the surfaces was approximately 300 mmols·m^−2^·s^−1^ (± 1 mmols·m^−2^·s^−1^). Tanks were exposed to a 10/14 h light-dark cycle. Microbial communities were allowed to grow until stabilization, which was determined by the exhibition of a structure and a composition similar to those of original mats^24^,^46^,^73^ (Supp Fig. S7). In addition, physicochemical variables related to mat activity, such as pH and DO concentration^46^, were relatively constant at this point during the illumination period, similar to natural conditions^74^,^75^.

### Experimental taphonomy

The experiment was conducted by using the African dwarf frog *Hymenochirus boettgeri* (Pipidae, Xenoanuran) as a model. Frogs were purchased from a research-specialized animal store and euthanized with tricaine mesylate (MS-222, 0.06%) diluted in TRIS (0.29%). Dead bodies (9 in each tank, N_total_ = 36) were laid on the surface of microbial mats (see below) with a 2.5 cm gap between the specimens. Individuals were randomly chosen (one per tank per time) and analysed at days 7, 15, 30, 120, 240, 540 and 1080 of the experiment (or until the decay state made manipulation impossible). Quantitative data were collected on the following morphometric variables: total body length, width at the base of the head, width of the pelvis, femur length, tibia length, and head, abdomen and thigh thickness. Measurements were taken with a digital calliper, and the carcasses were separated from the mat by cutting a thin layer of the sarcophagus under a stereo-microscope for measuring. In the controls, frogs were removed with a small spatula in order to minimize damage and cleaned by removing sediment particles with drops of water. The quantitative data were analysed using an ANOVA test to compare the loss in volume (determined by morphometric measurements) between the controls and frogs decaying in the mats over the entire time of the experiment. The null hypothesis tested, i.e., decay in microbial mats did not differ from the control decomposition, was rejected with a p-value ≤0.01. As the factor “time” had more than 2 groups, a comparison by pairs was conducted with a Bonferroni test.

Qualitative observations focused on swelling, colour (i.e., preservation of the original pigmentation), red colour (i.e., original colour shifting to red, which was observed during preliminary tests), visualization of the linea alba (i.e., a fibrous structure that is mostly composed of collagen connective tissue and crosses the midline of the abdomen in vertebrates^76^), presence of large amounts of exopolymeric substances (EPS), roughness of the skin, and grade of articulation. These features were ordinally scaled from 1 to 3. The data were collected at days 7, 15, 30, 120, and 240. However, advanced decay prevented measurement of the control at day 240. After day 240, the inclusion of the body in the mat was so intense that measurement was stopped. The data from the qualitative monitoring were analysed using Hierarchical Cluster Analysis to check resemblances (using between-group linkage as a clustering method-squared Euclidean distance) of variables between samples. The resulting Dendrogram described the sequence of changes during decay according to the presence or absence of mats. All statistical analyses were performed using the analytical software IBM SPSS Statistics 22.

### Scanning electron microscopy (SEM) and energy dispersive x-ray spectrometry (EDXS)

Electron microscopy was used to track occurrences of precipitates in the sarcophagus and inspect different aspects of the tissue and carcass integrity. For these analyses, samples of frogs were observed within their microbial sarcophagi. Because of the inherent features of the mat, in particular its dense EPS layer, chemicals used in sample preparation do not penetrate well inside the block. Consequently, a specific protocol for microbial mats was established after previous trials were conducted in our laboratory. Following these trials, the entire block was fixed in vacuum at room temperature during 64 h with 2% glutaraldehyde, dehydrated in ethanol at increasing concentrations (30%, 50%, 70%, 90%, 3 × 100%, 60 min each step) and lastly dried overnight at 37 °C. Prior to dehydration, samples were sectioned along a coronal plane. To obtain precise details, several parts, such as legs, were embedded in epoxy resin and polished with diamonds down to ¾ μm in size. All samples were then coated with gold. Images and analyses were collected in backscattered and secondary electron modes using a Hitachi S3000N operating at 15 kV, with a 60 μm aperture at a working distance of approximately 15 mm. Elemental compositions were determined using energy dispersive X-ray spectrometry (EDXS) with an Oxford Instruments INCAx-sight system.

## Additional Information

**How to cite this article**: Iniesto, M. *et al*. The Effect Of Microbial Mats In The Decay Of Anurans With Implications For Understanding Taphonomic Processes In The Fossil Record. *Sci. Rep.* **7**, 45160; doi: 10.1038/srep45160 (2017).

**Publisher's note:** Springer Nature remains neutral with regard to jurisdictional claims in published maps and institutional affiliations.

## Supplementary Material

Supplementary Information

## References

[b1] Murdock D. J. E., Gabbott S. E. & Purnell M. A. The impact of taphonomic data on phylogenetic resolution: Helenodora inopinata (Carboniferous, Mazon Creek Lagerstätte) and the onychophoran stem lineage. BMC Evol. Biol. 16, 19 (2016). 26801389 10.1186/s12862-016-0582-7 PMC4722706

[b2] Butler A. D., Cunningham J. A., Budd G. E. & Donoghue P. C. J. Experimental taphonomy of Artemia reveals the role of endogenous microbes in mediating decay and fossilization. Proc. Biol. Sci. 282, 20150476 (2015). 25972468 10.1098/rspb.2015.0476 PMC4455810

[b3] Gill-King H. In Forensic taphonomy: the postmortem fate of human remains (eds Sorg M. H. & Haglund W. D.) 93–108 (CRC Press, 1996).

[b4] Raff E. C., Villinski J., Turner F. R., Donoghue P. C. J. & Raff R. A. Experimental taphonomy shows the feasibility of fossil embryos. Proc. Natl. Acad. Sci. USA 103, 5846–5851 (2006). 16571655 10.1073/pnas.0601536103 PMC1416897

[b5] Raff E. C. . Embryo fossilization is a biological process mediated by microbial biofilms. Proc. Natl. Acad. Sci. USA 105, 19360–5 (2008). 19047625 10.1073/pnas.0810106105 PMC2614766

[b6] Raff R. A. . Microbial ecology and biofilms in the taphonomy of soft tissues. Palaios 29, 560–569 (2014).

[b7] Raff E. C. . Contingent interactions among biofilm-forming bacteria determine preservation or decay in the first steps toward fossilization of marine embryos. Evol. Dev. 15, 243–56 (2013). 23809699 10.1111/ede.12028

[b8] Briggs D. E. G. & Kear A. J. Fossilization of soft tissue in the laboratory. Science (80-). 259, 1439–42 (1993). 17801278 10.1126/science.259.5100.1439

[b9] Hof C. H. J. & Briggs D. E. G. Decay and mineralization of mantis shrimps (Stomatopoda; Crustacea); a key to their fossil record. Palaios 12, 420–438 (1997).

[b10] Sansom R. S., Gabbott S. E. & Purnell M. A. Non-random decay of chordate characters causes bias in fossil interpretation. Nature 463, 797–800 (2010). 20118914 10.1038/nature08745

[b11] Wilson P., Parry L. A., Vinther J. & Edgecombe G. D. Unveiling biases in soft-tissue phosphatization: Extensive preservation of musculature in the cretaceous (cenomanian) polychaete Rollinschaeta myoplena (annelida: amphinomidae). Palaeontology 59, 463–479 (2016).

[b12] Sansom R. S. Preservation and phylogeny of Cambrian ecdysozoans tested by experimental decay of Priapulus. Sci. Rep. 6, 32817 (2016). 27595908 10.1038/srep32817 PMC5011709

[b13] Briggs D. E. G. & Kear A. J. Decay of Branchiostoma: implicationes for soft-tissue preservation in conodonts and other primitive chordates. Lethaia 26, 275–287 (1994).

[b14] Wilby P. R., Briggs D. E. G., Bernier P. & Gaillard C. Role of microbial mats in the fossilization of soft tissues. Geology 24, 787 (1996).

[b15] Martin D., Briggs D. E. G. & Parkes R. J. Experimental mineralization of invertebrate eggs and the preservation of Neoproterozoic embryos. Geology 31, 39–42 (2003).

[b16] Martin D., Briggs D. E. G. & Parkes R. J. Decay and mineralization of invertebrate eggs. Palaios 20, 562–572 (2005).

[b17] Seilacher A. Begriff und bedeutung der Fossil-Lagerstätten. Neues Jarhb. für Geol. und Paläontologie 1, 34–39 (1970).

[b18] Seilacher A. . Sedimentological, ecological and temporal patterns of Fossil-Lagerstätten [and Discussion]. Philos. Trans. R. Soc. B Biol. Sci. 311, 5–24 (1985).

[b19] Marty D., Strasser A. & Meyer C. A. Formation and taphonomy of human footprints in microbial mats of present-day tidal-flat environments: implications for the study of fossil footprints. Ichnos 16, 127–142 (2009).

[b20] Cohen Y. & Rosenberg E. Microbial mats: physiological ecology of benthic microbial communities. (American Society for Microbiology, 1989).

[b21] Stolz J. F. In Microbial sediments (eds Riding R. E. & Awramik S. M.) 1–8 (Springer Berlin Heidelberg, 2000).

[b22] Wierzchos J., Berlanga M., Ascaso C. & Guerrero R. Micromorphological characterization and lithification of microbial mats from the Ebro Delta (Spain). Int. Microbiol. 9, 289–95 (2006). 17236163

[b23] Tomescu A. M. F., Klymiuk A. A., Matsunaga K. K. S., Bippus A. C. & Shelton G. W. K. In advances in environmental microbiology. Their world: a diversity of microbial environments (ed. Hurst C. J.), doi: 10.1007/978-3-319-28071-4_3 69–169 (Springer International Publishing) (2016).

[b24] Iniesto M., López-Archilla A. I., Fregenal-Martínez M. A., Buscalioni Á. D. & Guerrero M. C. Involvement of microbial mats in delayed decay: an experimental essay on fish preservation. Palaios 28, 56–66 (2013).

[b25] Chafetz H. S. & Buczynski C. Bacterially induced lithification of microbial mats. Palaios 7, 277–293 (1992).

[b26] Dupraz C. & Visscher P. T. Microbial lithification in marine stromatolites and hypersaline mats. Trends Microbiol. 13, 429–38 (2005). 16087339 10.1016/j.tim.2005.07.008

[b27] Dupraz C. . Processes of carbonate precipitation in modern microbial mats. Earth-Science Rev. 96, 141–162 (2009).

[b28] Decho A. W. & Kawaguchi T. In Fossil and recent biofilms: a natural history of life on Earth (eds Krumbein W. E., Paterson D. M. & Zavarzin G. A.), doi: 10.1007/978-94-017-0193-8_18 227–240 (Springer: Netherlands, 2003).

[b29] Iniesto M. . Preservation in microbial mats: mineralization by a talc-like phase of a fish embedded in a microbial sarcophagus. Front. Earth Sci. 3, (2015).

[b30] Iniesto M. . Involvement of microbial mats in early fossilization by decay delay and formation of impressions and replicas of vertebrates and invertebrates. Sci. Rep. 6, 1–12 (2016). 27162204 10.1038/srep25716 PMC4861970

[b31] Martín-Abad H. J. In Palaeobiology of the amiiform fishes from the Early Cretaceous of las hoyas 285–312 (Universidad Autónoma de Madrid, 2015).

[b32] Martín-Abad H. J. & Poyato-Ariza F. J. In Las Hoyas: A Cretaceous wetland. A multidisciplinary synthesis after 25 years of research on an exceptional fossil Lagerstätte from Spain (eds Poyato-Ariza F. J. & Buscalioni, ) 202–210 (Verlag Dr. Friedrich Pfeil, 2016).

[b33] Cambra-Moo Ó. & Buscalioni Á. D. Biostratinomic patterns in archosaur fossils: influence of morphological organization on dispersal. Journal of taphonomy 1, 247–296 (2003).

[b34] McNamara M. E. . Soft-tissue preservation in Miocene frogs from Libros, Spain: insights into the genesis tf decay microenvironments. Palaios 24, 104–117 (2009).

[b35] Mcnamara M. E. . Exceptionally preserved tadpoles from the Miocene of Libros, Spain: ecomorphological reconstruction and the impact of ontogeny upon taphonomy. Lethaia 43, 290–306 (2010).

[b36] Buscalioni Á. D. & Fregenal-Martínez M. A. A holistic approach to the palaeoecology of Las Hoyas Konservat-Lagerstätte (La Huérguina Formation, Lower Cretaceous, Iberian Ranges, Spain). J. Iber. Geol. 36, 297–326 (2010).

[b37] Gupta N. S. . Molecular taphonomy of macrofossils from the Cretaceous Las Hoyas Formation, Spain. Cretac. Res. 29, 1–8 (2008).

[b38] Báez A. M. Anurans from the Early Cretaceous Lagerstätte of Las Hoyas, Spain: New evidence on the Mesozoic diversification of crown-clade Anura. Cretac. Res. 41, 90–106 (2013).

[b39] Brito P. M. In The Crato fossils beds of Brazil (eds Martill D. M., Bechly G. & Loveridge R. F.) 429–443 (Cambridge University Press, 2007).

[b40] Leal M. E. C., Martill D. M. & Brito P. M. In The Crato fossils beds of Brazil (eds Martill D. M., Bechly G. & Loveridge R. F.) 444–451 (Cambridge University Press, 2007).

[b41] Ferber C. T. & Wells N. A. Paleolimnology and taphonomy of some fish deposits in ‘Fossil’ and ‘Uinta’ lakes of the Eocene river formation, Utah and Wyoming. Palaeogeogr. Palaeoclimatol. Palaeoecol. 117, 185–210 (1995).

[b42] Gómez R. O., Báez A. M. & Muzzopappa P. A new helmeted frog (Anura: Calyptocephalellidae) from an Eocene subtropical lake in northwestern Patagonia, Argentina. J. Vertebr. Paleontol. 31, 50–59 (2011).

[b43] Archer M., Godthelp H., Hand S. J. & Megirian D. Fossil mammals of Riversleigh, Northwestern Queensland: Preliminary overview of biostratigraphy, correlation and environmental change. Aust. Zool. 25, 29–65 (1989).

[b44] Allison P. A. & Briggs D. E. G. Exceptional fossil record: Distribution of soft-tissue preservation through the Phanerozoic. Geology 21, 527 (1993).

[b45] López-García Á., Martín-Abad H. J. & Cambra-Moo Ó. In Las Hoyas: A Cretaceous Wetland. A multidisciplinary synthesis after 25 years of research on an exceptional fossil Lagerstätte from Spain (eds Poyato-Ariza F. J. & Buscalioni Á. D.) 211–215 (Verlag Dr. Friedrich Pfeil, 2016).

[b46] Iniesto M. . The impact of microbial mats and their microenvironmental conditions in early decay of fish. Palaios 30, 792–801 (2015).

[b47] Cambra-Moo Ó., Buscalioni Á. D. & Delgado-Buscalioni R. An approach to the study of variations in early stages of Gallus gallus decomposition. J. Taphon. 6, 21–41 (2008).

[b48] Briggs D. E. G., Kear A. J., Martill D. M. & Wilby P. R. Phosphatization of soft-tissue in experiments and fossils. J. Geol. Soc. London. 150, 1035–1038 (1993).

[b49] Briggs D. E. G. In Fossil and recent biofilms: a natural history of life on Earth (eds Krumbein W. E., Paterson D. M. & Zavarzin G. A.) doi: 10.1007/978-94-017-0193-8_18 281–290 (Springer Netherlands, 2003).

[b50] Song Y., Hahn H. & Hoffmann E. In Chemical water and wastewater treatment (eds Hahn H., Hoffmann E. & Odegaard H.) 349–362 (IWA Publishing, 2002).

[b51] Recillas S. . Studies on the precipitation behavior of calcium phosphate solutions. J. Ceram. Process. Res. 13, 5–10 (2012).

[b52] Pace A. . Microbial and diagenetic steps leading to the mineralisation of Great Salt Lake microbialites. Sci. Rep. 6, 31495 (2016). 27527125 10.1038/srep31495 PMC4985759

[b53] Tarhan L. G., Hood A. vS, Droser M. L., Gehling J. G. & Briggs D. E. Exceptional preservation of soft-bodied Ediacara Biota promoted by silica-rich oceans. Geology 44, 951–954 (2016).

[b54] Alleon J. . Early entombment within silica minimizes the molecular degradation of microorganisms during advanced diagenesis. Chem. Geol. 437, 98–108 (2016).

[b55] Conlon J. M. & Mechkarska M. Host-Defense Peptides with Therapeutic Potential from Skin Secretions of Frogs from the Family Pipidae. Pharmaceuticals 7, 58–77 (2014). 24434793 10.3390/ph7010058 PMC3915195

[b56] Ali M. F., Soto A., Knoop F. C. & Conlon J. M. Antimicrobial peptides isolated from skin secretions of the diploid frog, Xenopus tropicalis (Pipidae). Biochim. Biophys. Acta-Protein Struct. Mol. Enzymol. 1550, 81–89 (2001). 10.1016/s0167-4838(01)00272-2 11738090

[b57] Gardner J. D. The Fossil Record of Tadpoles. Foss. Impr. 72, 17–44 (2016).

[b58] Kowal-Linka M. & Bodzioch A. Genesis of the Lower Triassic bonebeds from Gogolin (S Poland): The impact of microbial mats on trapping of vertebrate remains. Palaeogeogr. Palaeoclimatol. Palaeoecol. 466, 38–58 (2017).

[b59] Schweitzer M. H. & Horner J. R. Intravascular microstructures in trabecular bone tissues of Tyrannosaurus rex. Ann. Paléontologie 85, 179–192 (1999).

[b60] Trinajstic K., Marshall C., Long J. & Bifield K. Exceptional preservation of nerve and muscle tissues in Late Devonian placoderm fish and their evolutionary implications. Biol. Lett. 3, 197–200 (2007). 17284403 10.1098/rsbl.2006.0604 PMC2375963

[b61] McNamara M. . Organic preservation of fossil musculature with ultracellular detail. Proceedings. Biol. Sci. 277, 423–7 (2010). 10.1098/rspb.2009.1378 PMC2842642 19828545

[b62] Custer R. P. An atlas of the blood and bone marrow. (WB Saunders Company, 1974).

[b63] McNamara M. E. . High-fidelity organic preservation of bone marrow in ca. 10 Ma amphibians. Geology 34, 641 (2006).

[b64] Bertazzo S. . Fibres and cellular structures preserved in 75-million-year-old dinosaur specimens. Nat. Commun. 6, (2015). 10.1038/ncomms8352 PMC4468865 26056764

[b65] Cadena E. Microscopical and elemental FESEM and Phenom ProX-SEM-EDS analysis of osteocyte- and blood vessel-like microstructures obtained from fossil vertebrates of the Eocene Messel Pit, Germany. PeerJ 4, e1618 (2016). 26819855 10.7717/peerj.1618 PMC4727973

[b66] Armitage M. H. Preservation of Triceratops horridus tissue cells from the Hell Creek Formation, MT. Micros. Today 24, 18–23 (2016).

[b67] Schweitzer M. H., Moyer A. E. & Zheng W. Testing the hypothesis of biofilm as a source for soft tissue and cell-like structures preserved in dinosaur bone. PLoS One 11, e0150238 (2016). 26926069 10.1371/journal.pone.0150238 PMC4771714

[b68] Wilby P. R. The role of organic matrices in post-mortem phosphatization of soft-tissues. Kaupia 2, 99–113 (1993).

[b69] Báez A. M. in Las Hoyas: A Cretaceous wetland. A multidisciplinary synthesis after 25 years of research on an exceptional fossil Lagerstätte from Spain 143–150 (2016).

[b70] Sansom R. S. Bias and sensitivity in the placement of fossil taxa resulting from interpretations of missing data. Syst. Biol. 64, 256–266 (2015). 25432893 10.1093/sysbio/syu093 PMC4380037

[b71] Guerrero M. C., Balsa J., Pascual M., Martínez B. & Montes C. Caracterización limnológica de la Laguna Salada de Chiprana (Zaragoza, Espana) y sus comunidades de bacterias fototroficas. Limnetica 7, 83–96 (1991).

[b72] Vidondo B., Martínez B., Montes C. & Guerrero M. C. Physico-chemical characteristics of a permanent Spanish hypersaline lake: La Salada de Chiprana (NE Spain). Hydrobiologia 267, 113–125 (1993).

[b73] Jonkers H. M. . Structural and functional analysis of a microbial mat ecosystem from a unique permanent hypersaline inland lake: ‘La Salada de Chiprana’ (NE Spain). FEMS Microbiol. Ecol. 44, 175–189 (2003). 10.1016/S0168-6496(02)00464-6 19719635

[b74] Ludwig R., Al-Horani F. A., de Beer D. & Jonkers H. M. Photosynthesis-controlled calcification in a hypersaline microbial mat. Limnol. Oceanogr. 50, 1836–1843 (2005).

[b75] Ludwig R., Pringault O., de Wit R., de Beer D. & Jonkers H. M. Limitation of oxygenic photosynthesis and oxygen consumption by phosphate and organic nitrogen in a hypersaline microbial mat: a microsensor study. FEMS Microbiol. Ecol. 57, 9–17 (2006). 16819945 10.1111/j.1574-6941.2006.00109.x

[b76] Axer H., Keyserlingk D. G. V. & Prescher A. Collagen fibers in linea alba and rectus sheaths: General scheme and morphological aspects. J. Surg. Res. 96, 127–134 (2001). 11181006 10.1006/jsre.2000.6070

