# Supplementary material for: The Effect Of microbial Mats In The Decay Of Anurans With Implications For Understanding Taphonomic Processes In The Fossil Record
Source: Sci Rep. 2017 Mar 24;7:45160. doi: 10.1038/srep45160 (PMC5364532; doi:10.1038/srep45160)
Supplement: Supplementary Information [file srep45160-s1.pdf]

THE EFFECT OF MICROBIAL MATS IN THE DECAY OF ANURANS WITH IMPLICATIONS FOR UNDERSTANDING TAPHONOMIC PROCESSES IN THE FOSSIL RECORD

Iniesto M[ab*], Villalba I [a], Buscalioni AD[c], Guerrero MC[a], López-Archilla AI[a]

[a]*Department of Ecology, Universidad Autónoma de Madrid, 28049 Madrid, Spain.* [b]*Biogéosciences UMR6282, CNRS, Université Bourgogne Franche-Comté, 21000 Dijon, France;* [c]*Department of Biology, Universidad Autónoma de Madrid, 28049 Madrid Spain*

Keywords: brain mineralization, cell preservation, exceptional deposits, Pipidae, soft- tissue fossilization

*Correspondence to miguel.iniesto@gmail.com

Supplementary Figure S1: Sarcophagus formation and coverage of frog carcasses by mats.

(**A**) Frog over the mat at the beginning of the experiment. (**B**) Frog at day 12, with a distinguishable green-darkening under the carcass. (**C**) Frog partially covered at day 17. (**D**) Frog trapped by mat at day 28. (**E**) Thick sarcophagus covering the frog at day 43. (**F**) At day 60, the frog is embedded in the microbial community.

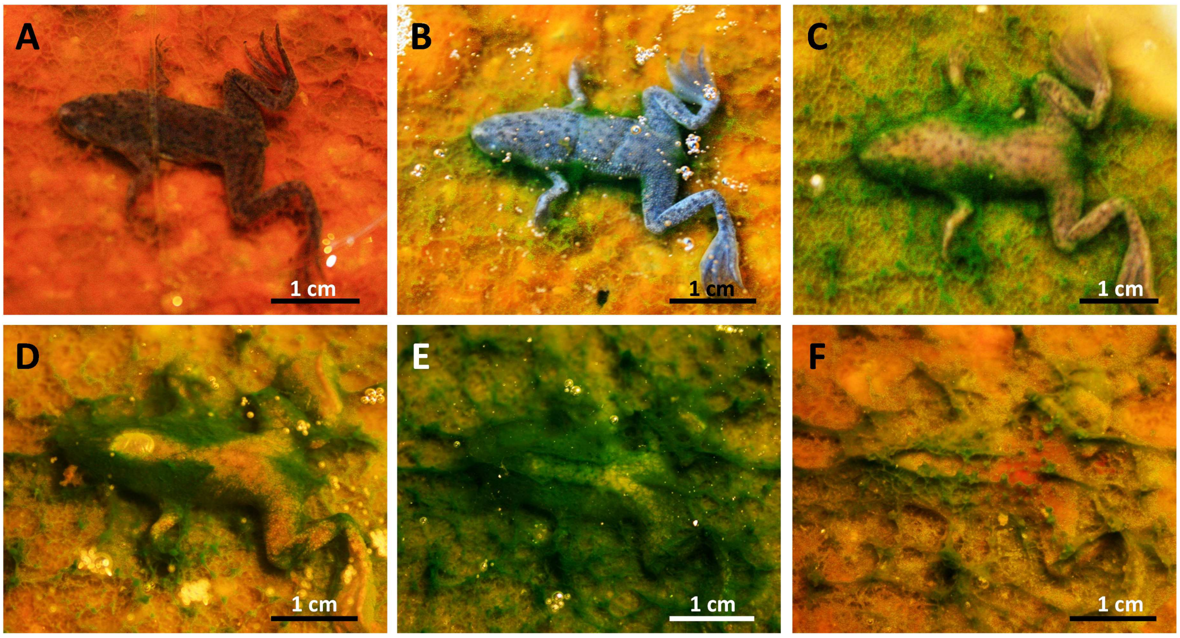

Supplementary Figure S2: Metric monitoring of bodies in the course of the experiment.

Loss of volume seemed to correlate with the kind of variable measured, being higher in the case of thickness (A), moderate for width (B) and lower for length (C). Data were represented in Scattered Plots by groups (A, B and C) in order to determine the existence of a trend line resuming the variation in volume. (A) Thickness: Data presents two different trend lines, corresponding with *Abdomen thickness* (blue) and *Head Thickness* (green marks). Equations showed that the loss in thickness was higher in those structures with more flesh such as the thigh, while bony structures such as the head exhibit a minor drop. *Thigh thickness* (red marks) did not show any trend line, likely due to the initial swelling. (B) In the case of Width, two trend lines appeared which corresponded with the two variables measured: *Head* (blue) and *Hip* (red) width. It is remarkable that reduction in width is smaller in those structures made mainly by bone such as the head. (C) Length's reduction was more similar between variables, and one single trend line summarized the whole data set (i.e. *Femur* (blue), *Tibia* (red) and *Total Body* (green). However, reduction was smaller than in other variables, most likely due to bones that are less responsive to decay.

A. Thickness

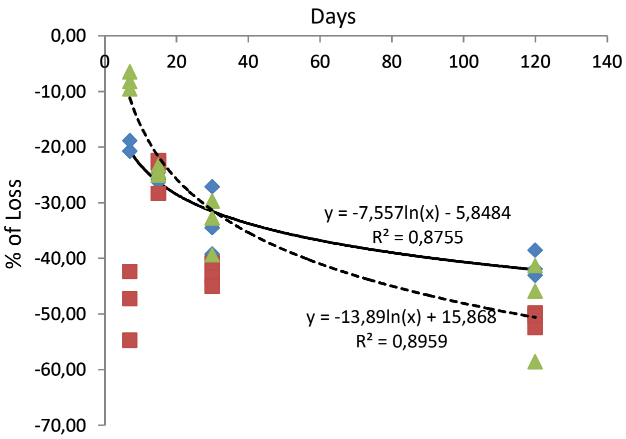

B. Width

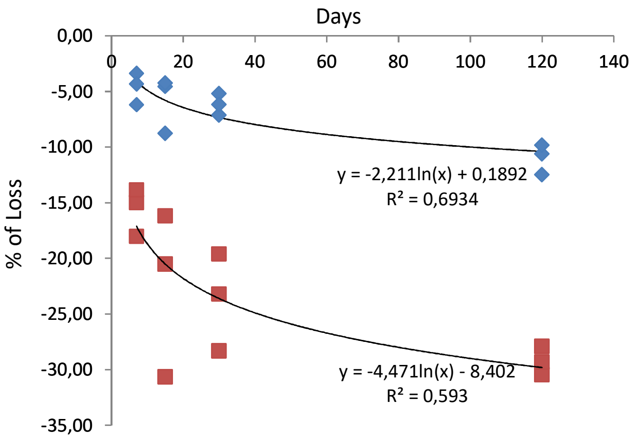

C. Lenght

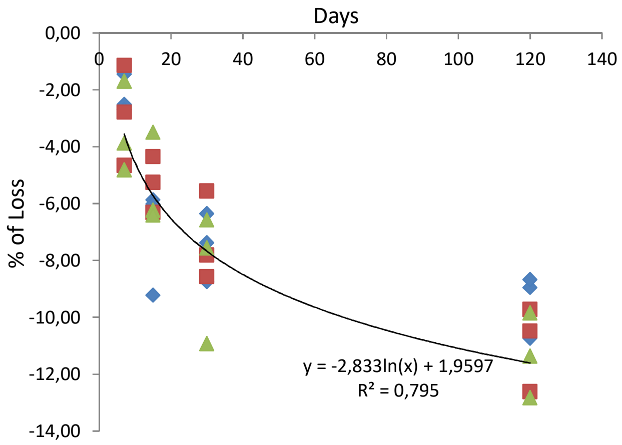

Supplementary Figure S3: ***Hymenochirus boettgeri*** **after its removal from the microbial sarcophagus at day 120.**

It is noticeable that specimen maintain the complete articulation of limbs and head while being manipulated for measurement. In addition, carcass shows an evident flattening.

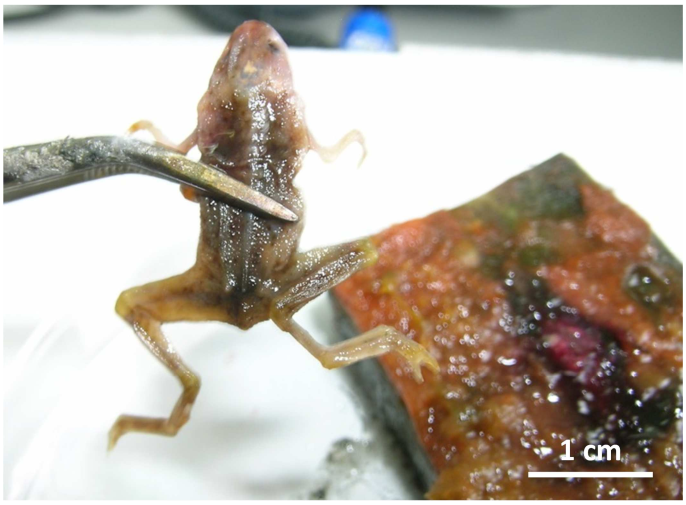

Supplementary Figure S4: State of articulation and soft tissue preservation of *Hymenochirus boettgeri* inside the microbial sarcophagus.

(**A**) Knee of a frog after 120 days in the mat. (**B**) Detail of the foot at day 120, where nails are still preserved. (**C**) Knee at day 240. (**D**) Foot of the frog at day 240 with nails still present. (**E**) Ankle of the frog at day 540 observed with SEM. The skin is still preserved. Its smoothness let the detection of muscle and tendons beneath it. (**F**) Section of completely articulated vertebrae (v) with the skin (sk) covering bones. mm: microbial mat; sed: sediment.

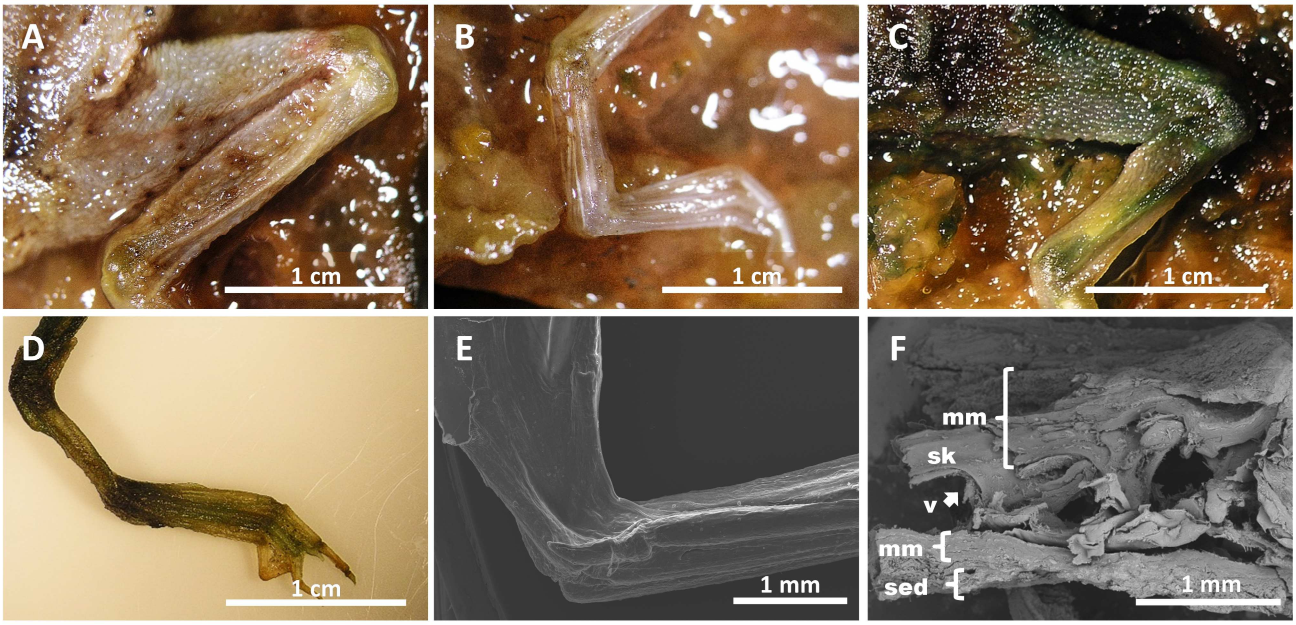

Supplementary Figure S5: Observation with SEM of the external condition of bones at day 1080 (3 years).

(**A**) Section of a vertebra still covered by the skin at day 1080. Where the skin (sk) is absent (the edge is pointed by the arrow), the surface of the bone (b) can be observed. The absence of the skin is a consequence of sectioning. (**B**) Detail of the femur surface.

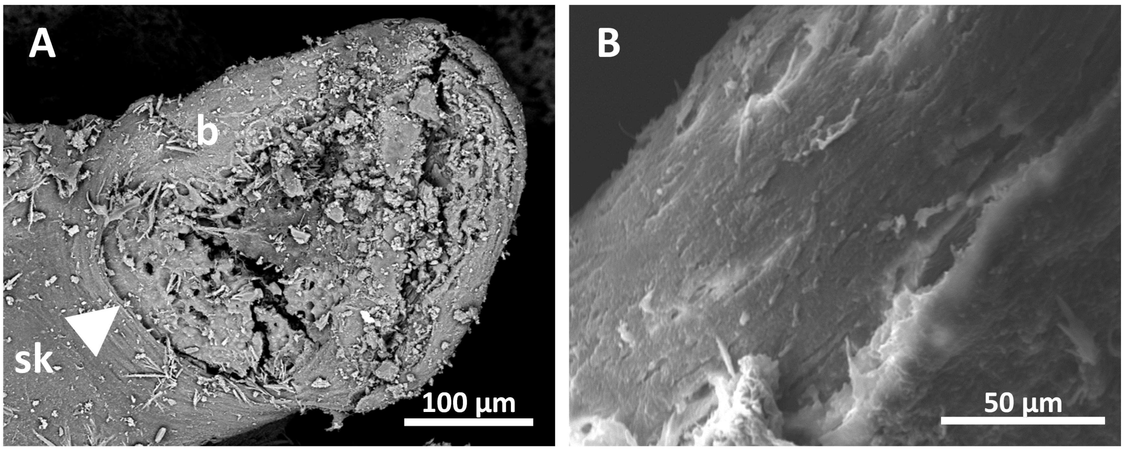

Supplementary Figure S6: Head of a frog after 120 days inside the sarcophagus.

Both tegument and bone have become slightly transparent and the midbrain (br) can be observed through them. Despite the time in tank, eyes (e) are still preserved and skin (sk) is not pierced nor broken. In addition, the original pattern of pigmentation (p) is preserved.

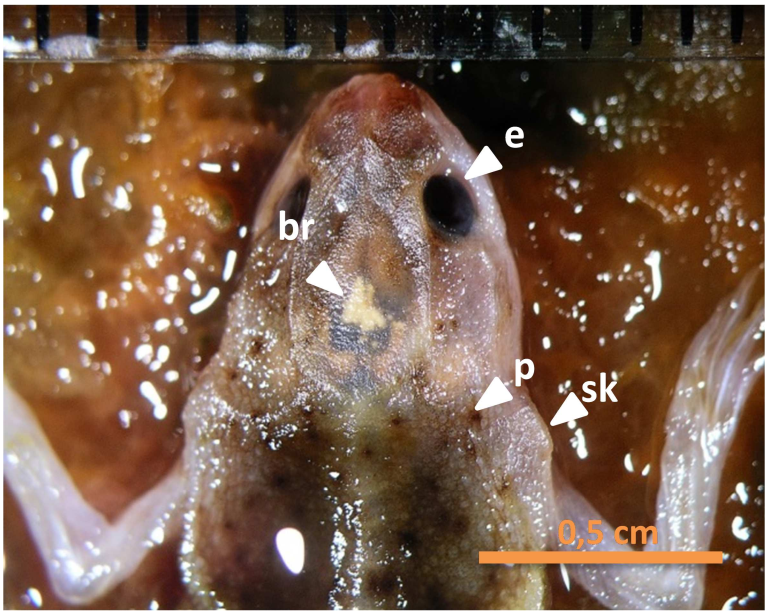

Supplementary Figure S7: Natural microbial mats recollected at Chiprana Lake (A and B) and microbial mats grown in controlled conditions (C and D).

(**A**) Profile section of a natural microbial mat collected at Chiprana Lake. The green line highlights the active zones, where "green, red and black layers" can be found. The predominant populations are cyanobacteria, purple anoxigenic photobacteria, and organo- and lithotrophic bacteria respectively. These strata are built up over former active layers. (**B**) Pinnacles (arrows) are characteristic structures of mats dominated by cyanobacteria. (**C**) Stabilized mat after development in laboratory. Its three characteristic layers can be easily observed. (**D**) Microbial pinnacles are also present in our experimental mats, which were assumed as a sign of maturity and stabilization.

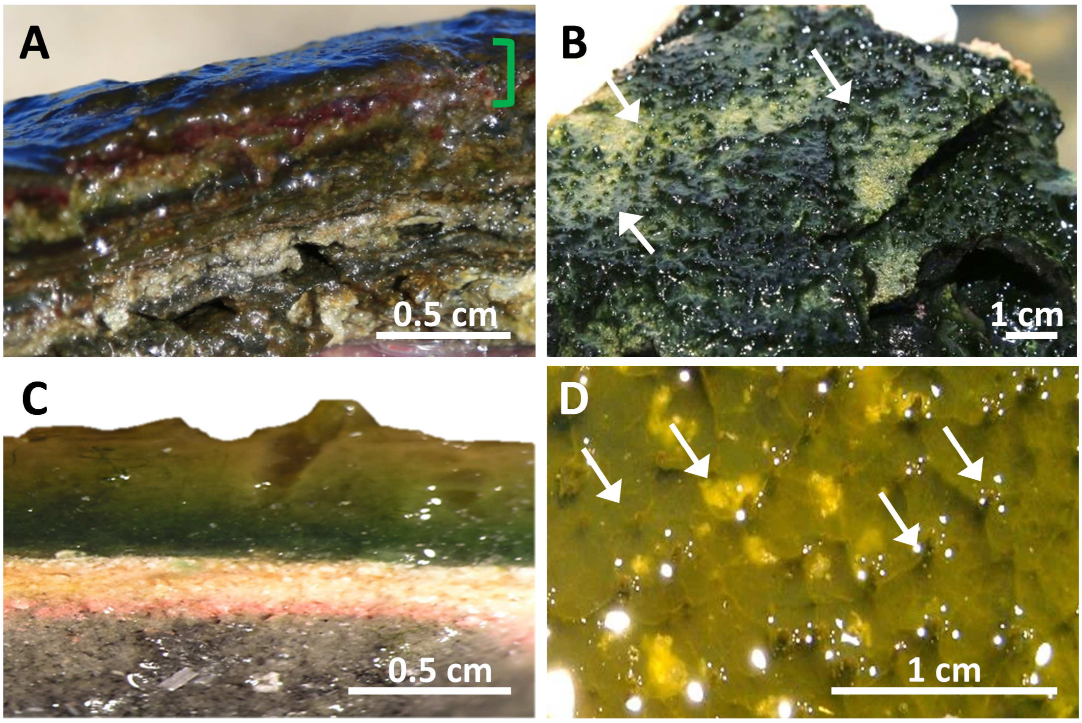

Supplementary Table S1: Data matrix for Hierarchical Cluster Test based on changes showed by decaying frogs in the course of the experiment.

Sequence of changes observed during the process of decomposition of frogs in non-mat control sediment and tanks with microbial mats. Initially, both were swelled (first 7 days), although bodies in mat samples recovered slowly their thickness on day 15. The longer persistence of the swelling in mat samples was consequent with the state of the integument. Controls presented at day 15 a loss of skin rugosity (likely pierced), quite disarticulation, and deformation. Thus, the accumulation of gases inside the body was avoided and volume returned to base values. During the swelling-time, the observation of the *linea alba* was difficult. In addition, a thin heterotrophic veil grew rapidly over controls, supporting the statement of the active decay. Mat samples did not show any deformation nor disarticulation along the experiment (Fig. 2 and 4), and a thick layer of mat covered the bodies. These features were ordinal scaled from 1 to 3. 0 = no changes observed; 1 = minor changes observed -i.e. structure is maintained but a few changes can be detected-; 2 = moderate changes; 3 = major changes observed -i.e. element is completely decayed or reduced to disarticulated rests-). T1, T2 and T3 correspond to tanks with microbial mats while C is the control sediment tank.

Time (days)	Tank	Swelling	Colour	Red colour	*Linea alba*	EPS	Skin	Articulation
**7**	T1	3	0	3	2	0	3	0
**7**	T2	3	0	3	2	0	3	0
**7**	T3	3	0	3	2	0	3	0
**7**	C	3	0	3	2	2	3	0
**15**	T1	2	0	2	3	1	2	0
**15**	T2	3	0	2	3	1	3	0
**15**	T3	2	0	1	2	1	2	0
**15**	C	0	0	1	1	3	1	1
**30**	T1	0	0	0	1	2	0	0
**30**	T2	3	0	0	3	3	3	0
**30**	T3	0	0	0	1	2	0	0
**30**	C	0	0	0	0	3	0	1
**120**	T1	0	0	1	3	3	0	0
**120**	T2	0	0	0	3	3	0	0
**120**	T3	0	0	0	3	3	0	0
**120**	C	0	0	0	3	3	0	3
**242**	T1	0	3	0	3	3	0	0
**242**	T2	0	3	0	3	3	0	0
**242**	T3	0	3	0	3	3	0	0

Supplementary Table S2: Statistical analysis using the Mann-Witney Wilcox test to compare differences size reduction between frogs into microbial mats and control frogs over sediment.

- Main null hypothesis tested: "decay (i.e. loss of size) in microbial mats did not show differences with control decomposition"
- Other null hypothesis tested: "decay process (i.e. loss of size) did not show differences over time"

Differences are considered significant (in bold) at a p-value< 0.01; df = degrees of freedom; F = Snedecor distribution.

Factor	Morphometric parameter	df	F	Sig.
**Time**	Total Lenght	3	46,548	**0,000**
	Head Width	3	14,383	**0,001**
	Hip Width	3	7,266	0,011
	Femur Lenght	3	42,561	**0,000**
	Fibula Lenght	3	28,608	**0,000**
	Head Thickness	3	40,188	**0,000**
	Abdomen Thickness	3	29,806	**0,000**
	Thigh Thickness	3	49,013	**0,000**
**Tank**	Total Lenght	1	51,804	**0,000**
	Head Width	1	339,116	**0,000**
	Hip Width	1	24,049	**0,001**
	Femur Lenght	1	87,994	**0,000**
	Fibula Lenght	1	44,427	**0,000**
	Head Thickness	1	129,707	**0,000**
	Abdomen Thickness	1	65,089	**0,000**
	Thigh Thickness	1	82,093	**0,000**

Supplementary Table S3: Bonferroni test for time factor showing differences between groups

Differences are considered significant (in bold) at a p-value< 0.01

Morphometric parameter	(I) Time 1	Vs (J) Time 2	Mean differences (I-J)	Sig.	Confidence interval (up to 95%) Upper limit	Lower limit
**Total Lenght**	7	15	5,7025(*)	**0,002**	2,5137	8,8913
		30	8,4025(*)	**0,000**	5,2137	11,5913
		120	7,0167(*)	**0,001**	3,5724	10,4609
	15	7	-5,7025(*)	**0,002**	-8,8913	-2,5137
		30	2,7000	0,111	-0,4888	5,8888
		120	1,3142	1,000	-2,1301	4,7584
	30	7	-8,4025(*)	**0,000**	-11,5913	-5,2137
		15	-2,7000	0,111	-5,8888	0,4888
		120	-1,3858	1,000	-4,8301	2,0584
	120	7	-7,0167(*)	**0,001**	-10,4609	-3,5724
		15	-1,3142	1,000	-4,7584	2,1301
		30	1,3858	1,000	-2,0584	4,8301
**Head Width**	7	15	2,1350	0,656	-1,9885	6,2585
		30	3,7950	0,075	-0,3285	7,9185
		120	2,2542	0,698	-2,1997	6,7080
	15	7	-2,1350	0,656	-6,2585	1,9885
		30	1,6600	1,000	-2,4635	5,7835
		120	0,1192	1,000	-4,3347	4,5730
	30	7	-3,7950	0,075	-7,9185	0,3285
		15	-1,6600	1,000	-5,7835	2,4635
		120	-1,5408	1,000	-5,9947	2,9130
	120	7	-2,2542	0,698	-6,7080	2,1997
		15	-0,1192	1,000	-4,5730	4,3347
		30	1,5408	1,000	-2,9130	5,9947
**Hip Width**	7	15	7,4675	0,279	-3,5732	18,5082
		30	10,7225	0,058	-0,3182	21,7632
		120	11,0250	0,074	-0,9003	22,9503
	15	7	-7,4675	0,279	-18,5082	3,5732
		30	3,2550	1,000	-7,7857	14,2957
		120	3,5575	1,000	-8,3678	15,4828
	30	7	-10,7225	0,058	-21,7632	0,3182
		15	-3,2550	1,000	-14,2957	7,7857
		120	0,3025	1,000	-11,6228	12,2278

	120	7	-11,0250	0,074	-22,9503	0,9003
		15	-3,5575	1,000	-15,4828	8,3678
		30	-0,3025	1,000	-12,2278	11,6228
**Femur Lenght**	7	15	4,4350(*)	0,017	0,7894	8,0806
		30	8,0650(*)	**0,000**	4,4194	11,7106
		120	7,6275(*)	**0,001**	3,6898	11,5652
	15	7	-4,4350(*)	0,017	-8,0806	-0,7894
		30	3,6300	0,051	-0,0156	7,2756
		120	3,1925	0,135	-0,7452	7,1302
	30	7	-8,0650(*)	**0,000**	-11,7106	-4,4194
		15	-3,6300	0,051	-7,2756	0,0156
		120	-0,4375	1,000	-4,3752	3,5002
	120	7	-7,6275(*)	**0,001**	-11,5652	-3,6898
		15	-3,1925	0,135	-7,1302	0,7452
		30	0,4375	1,000	-3,5002	4,3752
**Fibula Lenght**	7	15	3,2700	0,189	-1,0997	7,6397
		30	8,1800(*)	**0,001**	3,8103	12,5497
		120	7,4567(*)	**0,003**	2,7368	12,1765
	15	7	-3,2700	0,189	-7,6397	1,0997
		30	4,9100(*)	0,027	0,5403	9,2797
		120	4,1867	0,090	-0,5332	8,9065
	30	7	-8,1800(*)	**0,001**	-12,5497	-3,8103
		15	-4,9100(*)	0,027	-9,2797	-0,5403
		120	-0,7233	1,000	-5,4432	3,9965
	120	7	-7,4567(*)	**0,003**	-12,1765	-2,7368
		15	-4,1867	0,090	-8,9065	0,5332
		30	0,7233	1,000	-3,9965	5,4432
**Head Thickness**	7	15	8,6600(*)	0,038	0,4400	16,8800
		30	18,7100(*)	**0,000**	10,4900	26,9300
		120	17,5283(*)	**0,001**	8,6497	26,4070
	15	7	-8,6600(*)	0,038	-16,8800	-0,4400
		30	10,0500(*)	0,017	1,8300	18,2700
		120	8,8683	0,050	-0,0103	17,7470
	30	7	-18,7100(*)	**0,000**	-26,9300	-10,4900
		15	-10,0500(*)	0,017	-18,2700	-1,8300
		120	-1,1817	1,000	-10,0603	7,6970
	120	7	-17,5283(*)	**0,001**	-26,4070	-8,6497
		15	-8,8683	0,050	-17,7470	0,0103
		30	1,1817	1,000	-7,6970	10,0603
**Abdomen Thickness**	7	15	-13,7525(*)	**0,005**	-22,9598	-4,5452
		30	7,0375	0,173	-2,1698	16,2448
		120	5,0133	0,705	-4,9317	14,9583
	15	7	13,7525(*)	**0,005**	4,5452	22,9598

		30	20,7900(*)	**0,000**	11,5827	29,9973
		120	18,7658(*)	**0,001**	8,8208	28,7108
	30	7	-7,0375	0,173	-16,2448	2,1698
		15	-20,7900(*)	**0,000**	-29,9973	-11,5827
		120	-2,0242	1,000	-11,9692	7,9208
	120	7	-5,0133	0,705	-14,9583	4,9317
		15	-18,7658(*)	**0,001**	-28,7108	-8,8208
		30	2,0242	1,000	-7,9208	11,9692
**Thigh Thickness**	7	15	22,4800(*)	**0,002**	9,7028	35,2572
		30	32,0350(*)	**0,000**	19,2578	44,8122
		120	36,8792(*)	**0,000**	23,0782	50,6802
	15	7	-22,4800(*)	**0,002**	-35,2572	-9,7028
		30	9,5550	0,189	-3,2222	22,3322
		120	14,3992(*)	0,040	0,5982	28,2002
	30	7	-32,0350(*)	**0,000**	-44,8122	-19,2578
		15	-9,5550	0,189	-22,3322	3,2222
		120	4,8442	1,000	-8,9568	18,6452
	120	7	-36,8792(*)	**0,000**	-50,6802	-23,0782
		15	-14,3992(*)	0,040	-28,2002	-0,5982
		30	-4,8442	1,000	-18,6452	8,9568
